# Supplementary material for: W546 stacking disruption traps the human porphyrin transporter ABCB6 in an outward-facing transient state
Source: Commun Biol. 2023 Sep 21;6:960. doi: 10.1038/s42003-023-05339-3 (PMC10514269; doi:10.1038/s42003-023-05339-3)
Supplement: Supplementary file 2 — Supplementary Information [file 42003_2023_5339_MOESM2_ESM.pdf]

Supplementary Information

for

W546 stacking disruption traps the human porphyrin transporter ABCB6 in an outward-facing transient state

Sang Soo Lee[1], Jun Gyou Park[1], Eunhong Jang[1], Seung Hun Choi[1], Subin Kim[1], Ji Won Kim[2] & Mi Sun Jin*

[1]School of Life Sciences, GIST, 123 Cheomdan-gwagiro, Buk-gu, Gwangju 61005, Republic of Korea, [2]Department of Life Sciences, POSTECH, Nam-gu, Pohang 37673, Republic of Korea

Correspondence to: misunjin@gist.ac.kr

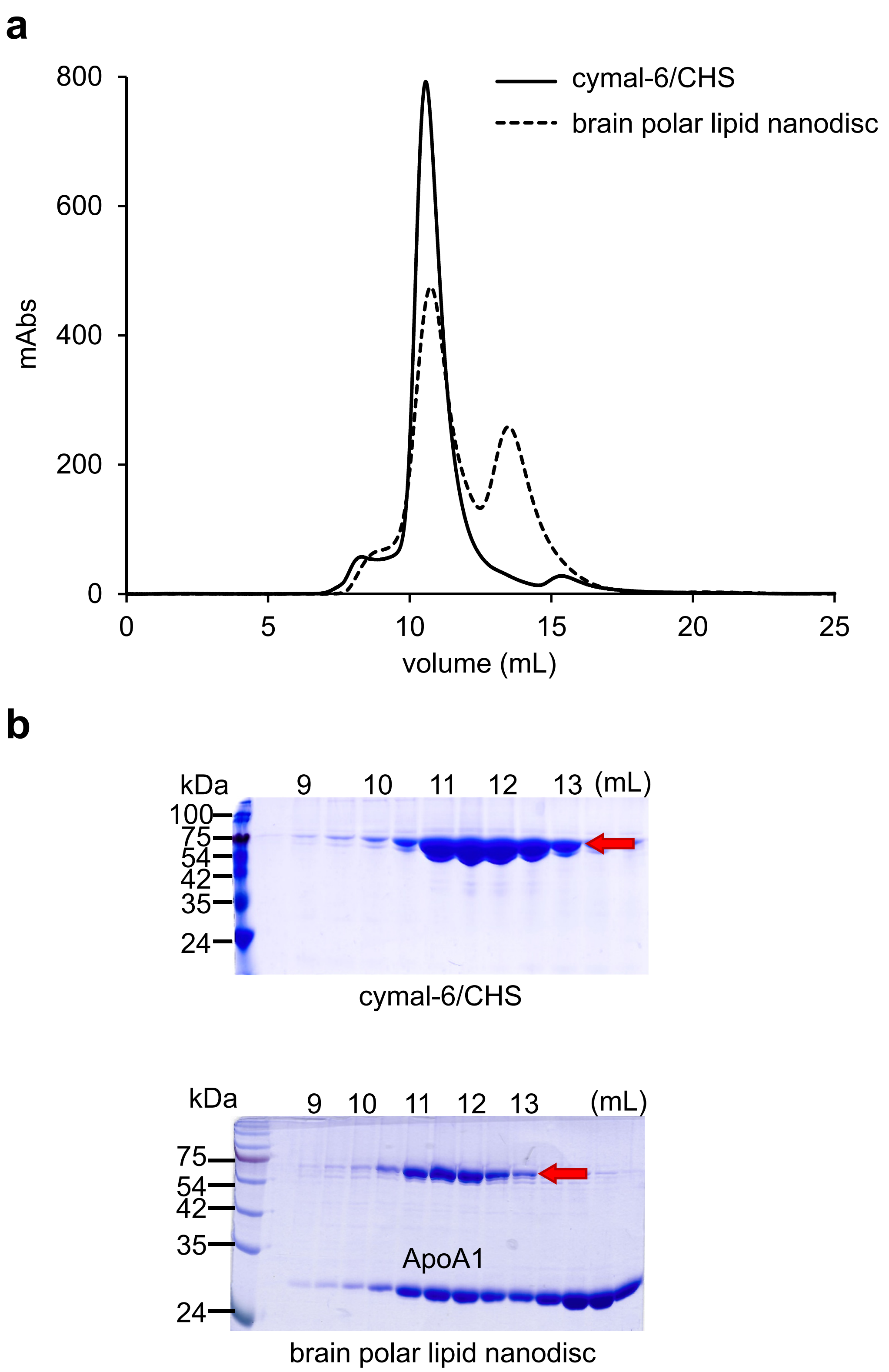

Supplementary Figure 1. Size-exclusion chromatography profile of hABCB6^{core} and SDS-PAGE analysis. (a) Elution profiles of hABCB6^{core} protein in detergent micelles or nanodiscs composed of porcine brain polar lipids using a Superdex Increase 200 10/300 GL column. (b) SDS-PAGE analysis of fractions from size exclusion chromatography. Bands of hABCB6^{core} protein are indicated by red arrows.

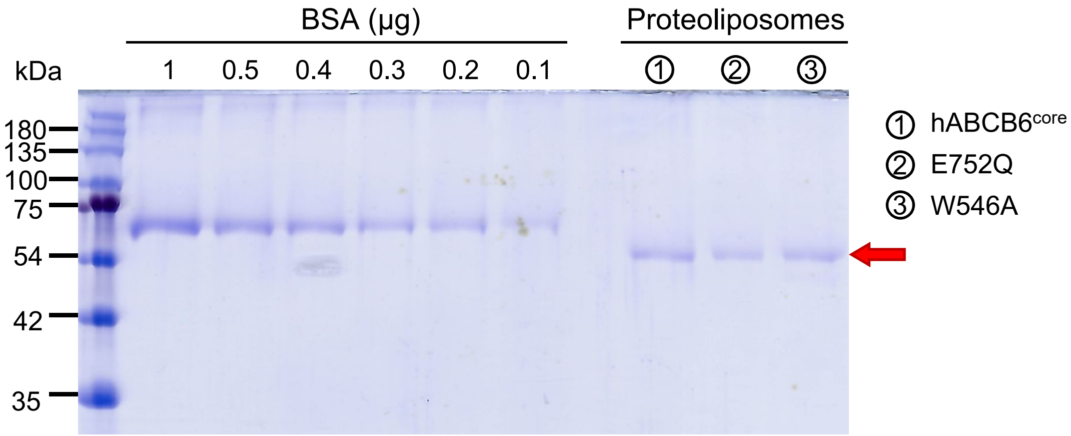

Supplementary Figure 2. SDS-PAGE analysis of proteoliposomes. Bovine serum albumin (BSA) (0.1–1 mg) and protein-loaded liposomes were separated by 12% (w/v) SDS-PAGE. The amounts of protein in liposomes were calculated using BSA calibration. The SDS-PAGE results show that ~1.8 mg of protein was reconstituted in each liposome. The gel is one representative result from at least two independent experiments.

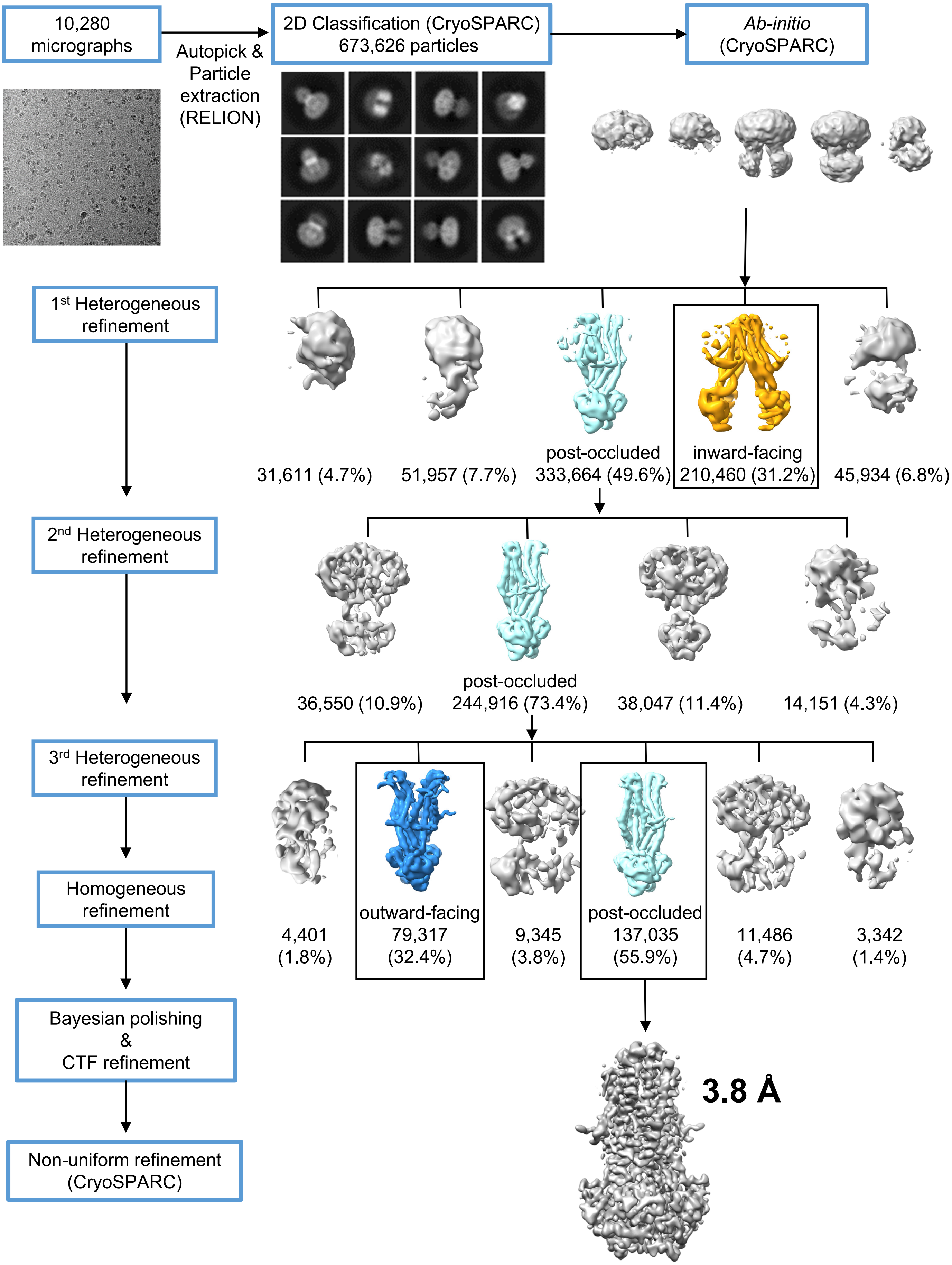

Supplementary Figure 3. Cryo-EM data processing pipeline employed for detergent-purified hABCB6^{core} in the presence of CPIII and Mg^{2+}/ADP·VO_4. The data processing workflow is presented and major 3D classes shown in Fig. 2b are in boxes.

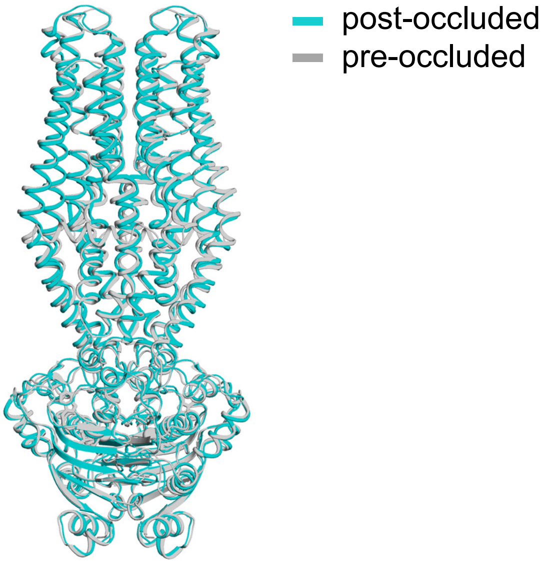

Supplementary Figure 4. Structural comparison of the pre- and post-occluded conformations of hABCB6^{core}. The overall structures of ATP-bound (pre-occluded, PDB ID 7EKL) and ADP·VO_4-bound (post-occluded, Fig. 4a) forms are superimposed.

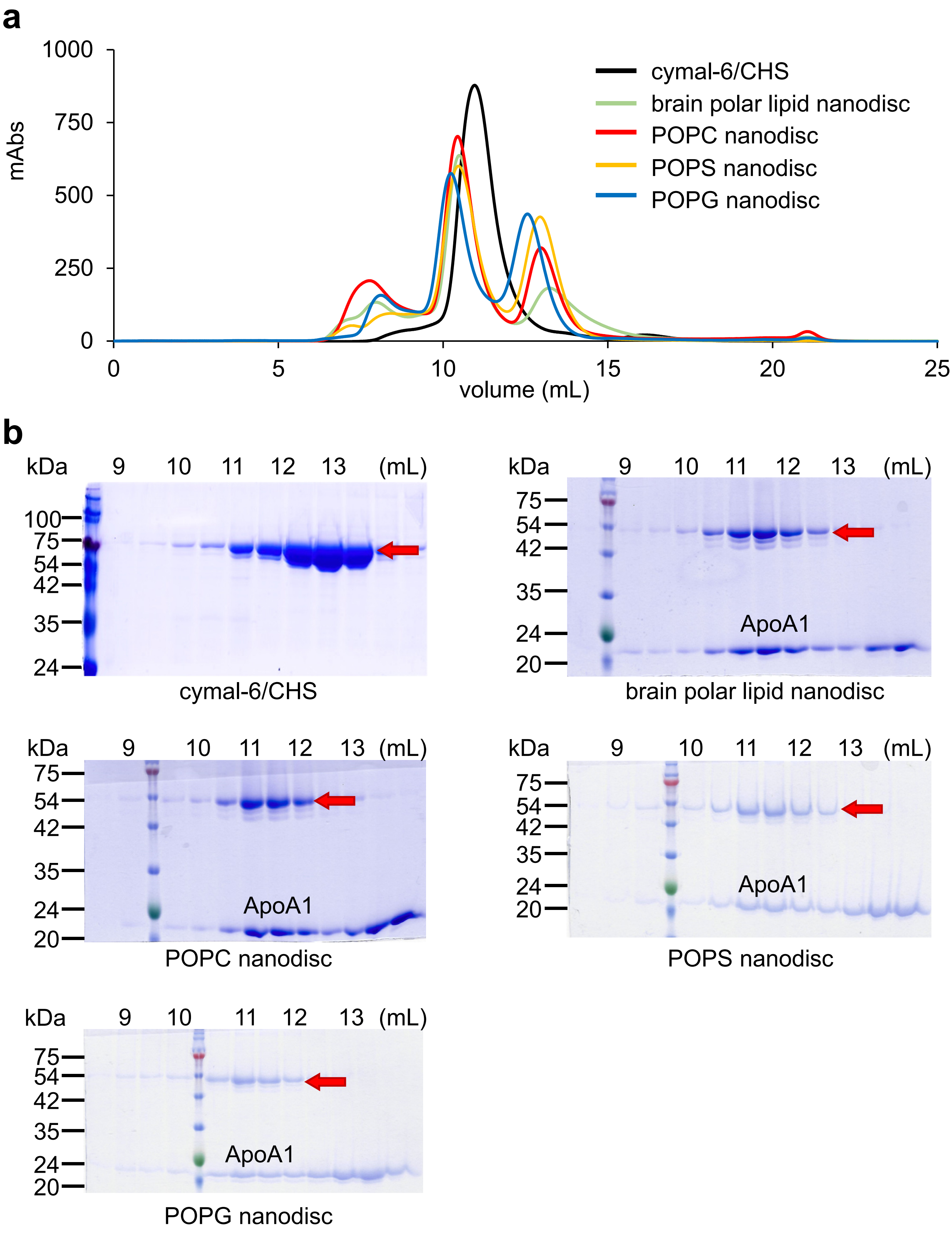

Supplementary Figure 5. Size exclusion chromatography profile and SDS-PAGE analysis of the W546A mutant. (a) Superdex Increase 200 10/300 GL column elution profiles of the W546A protein in detergent micelles or nanodiscs reconstituted using various lipids. (b) SDS-PAGE analysis of size exclusion chromatography fractions. Bands corresponding to the W546A protein are indicated by red arrows.

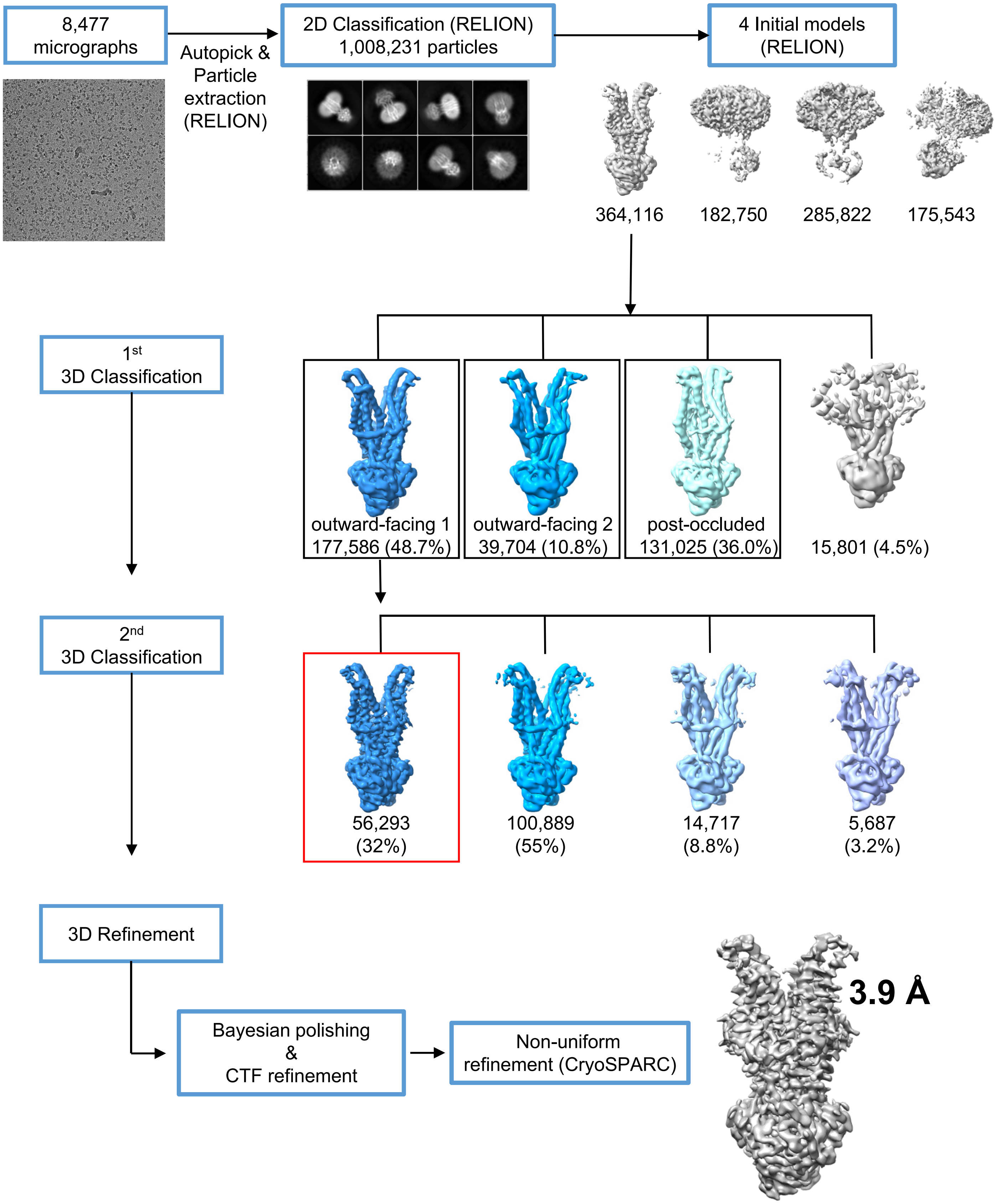

Supplementary Figure 6. Cryo-EM data processing pipeline employed for Mg^{2+}/ADP·VO_4-bound, outward-facing hABCB6^{core}-W546A in detergent micelles. The data processing workflow is presented and major 3D classes shown in Fig. 4b are in black boxes. The best 3D class subjected to the final 3D reconstruction is in a red box. Please see the Methods section for details.

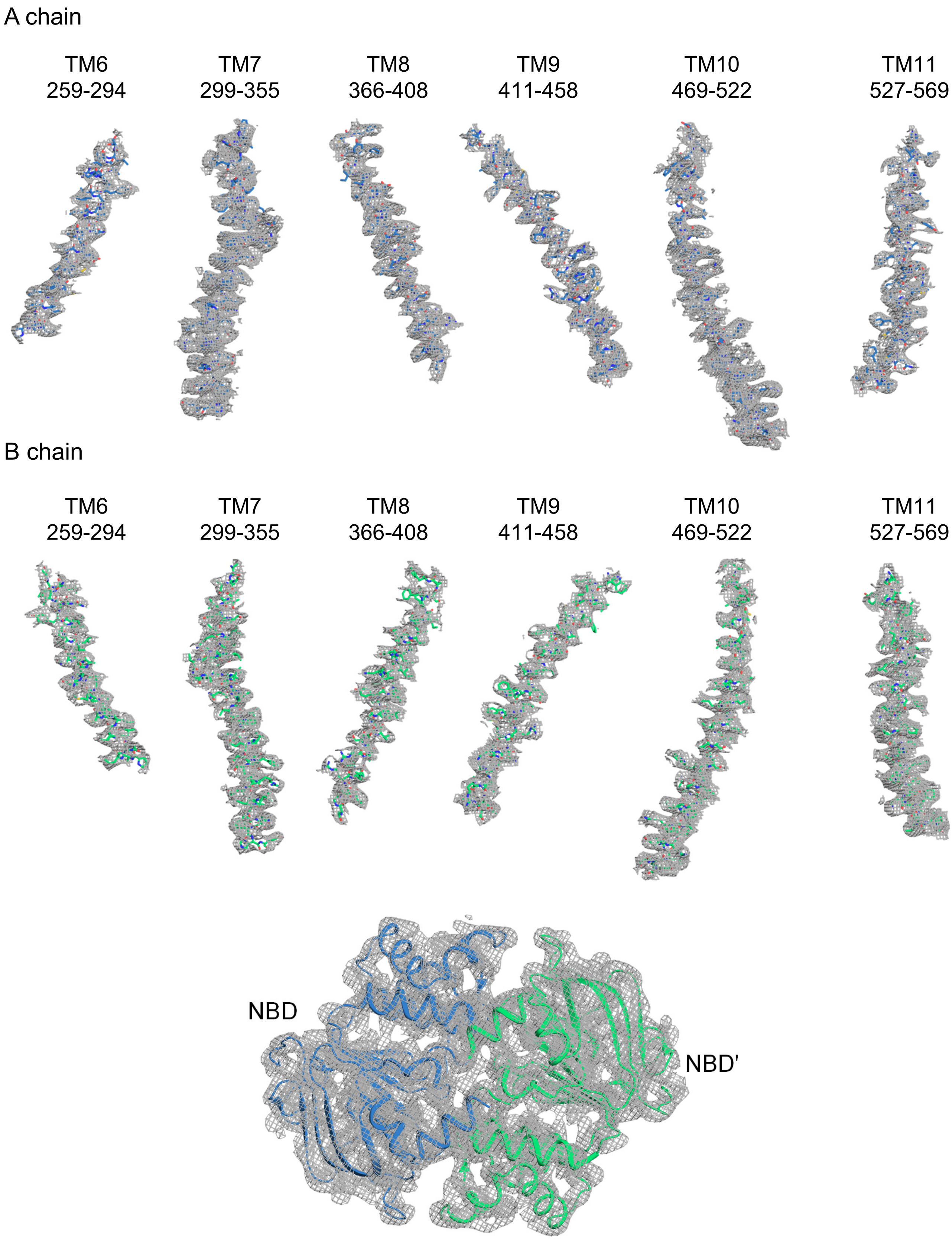

Supplementary Figure 7. Cryo-EM maps of outward-facing hABCB6^{core}-W546A in complex with Mg^{2+}/ADP·VO_4. Amino acid residues in TMDs are shown, while NBDs are depicted as cartoons. The map is contoured at the 4 σ level.

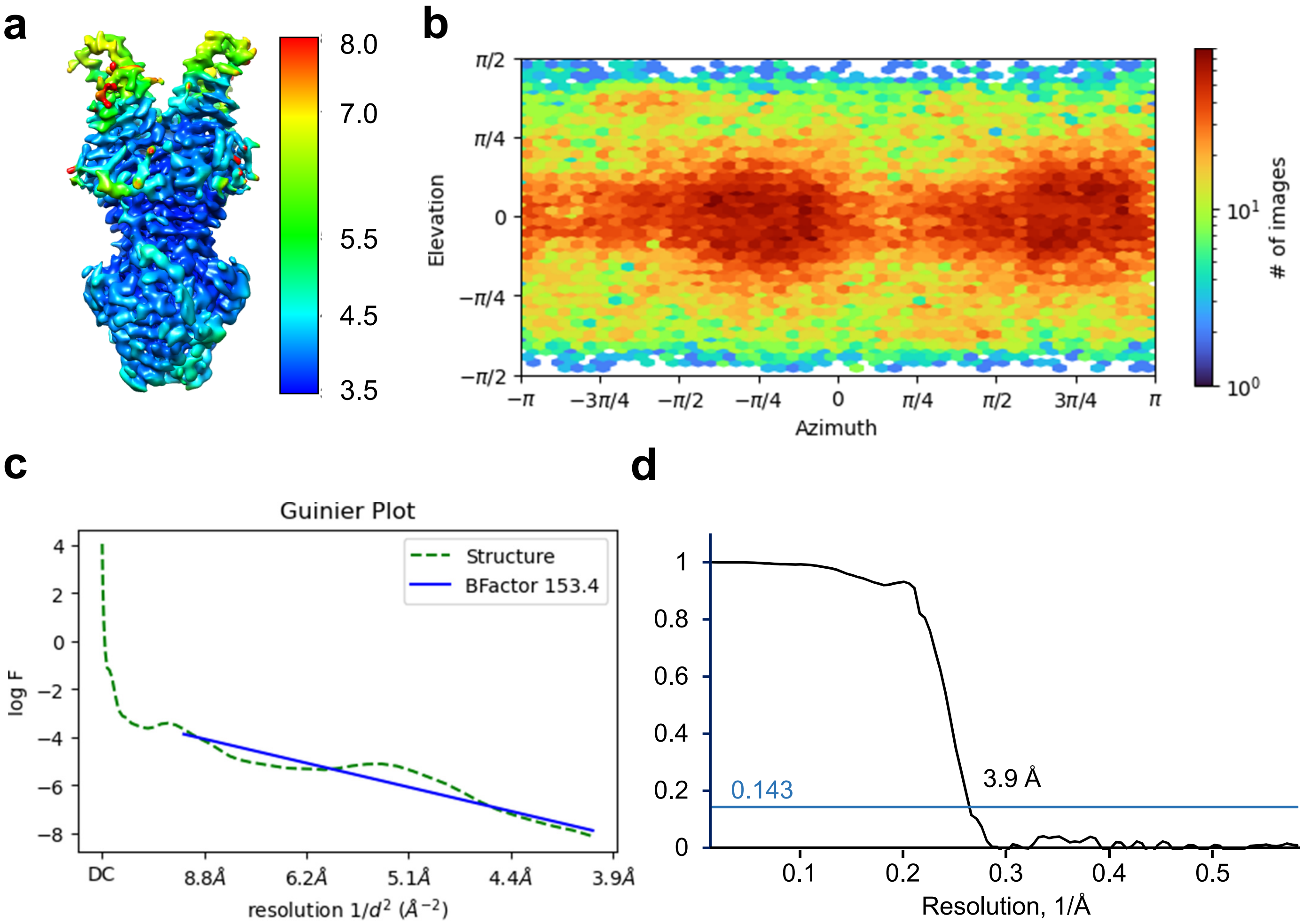

Supplementary Figure 8. Cryo-EM data quality analysis of outward-facing hABCB6^{core}-W546A in complex with Mg^{2+}/ADP·VO_4. (a) Local resolution of outward-facing hABCB6^{core}-W546A from 3.5 Å (blue) to 8.0 Å (red). (b) Particle orientation distributions in the final 3D reconstruction. (c) Guinier plot for global B-factor estimation. (d) Gold-standard FSC curve. The blue line represents the 0.143 FSC cut-off criterion.

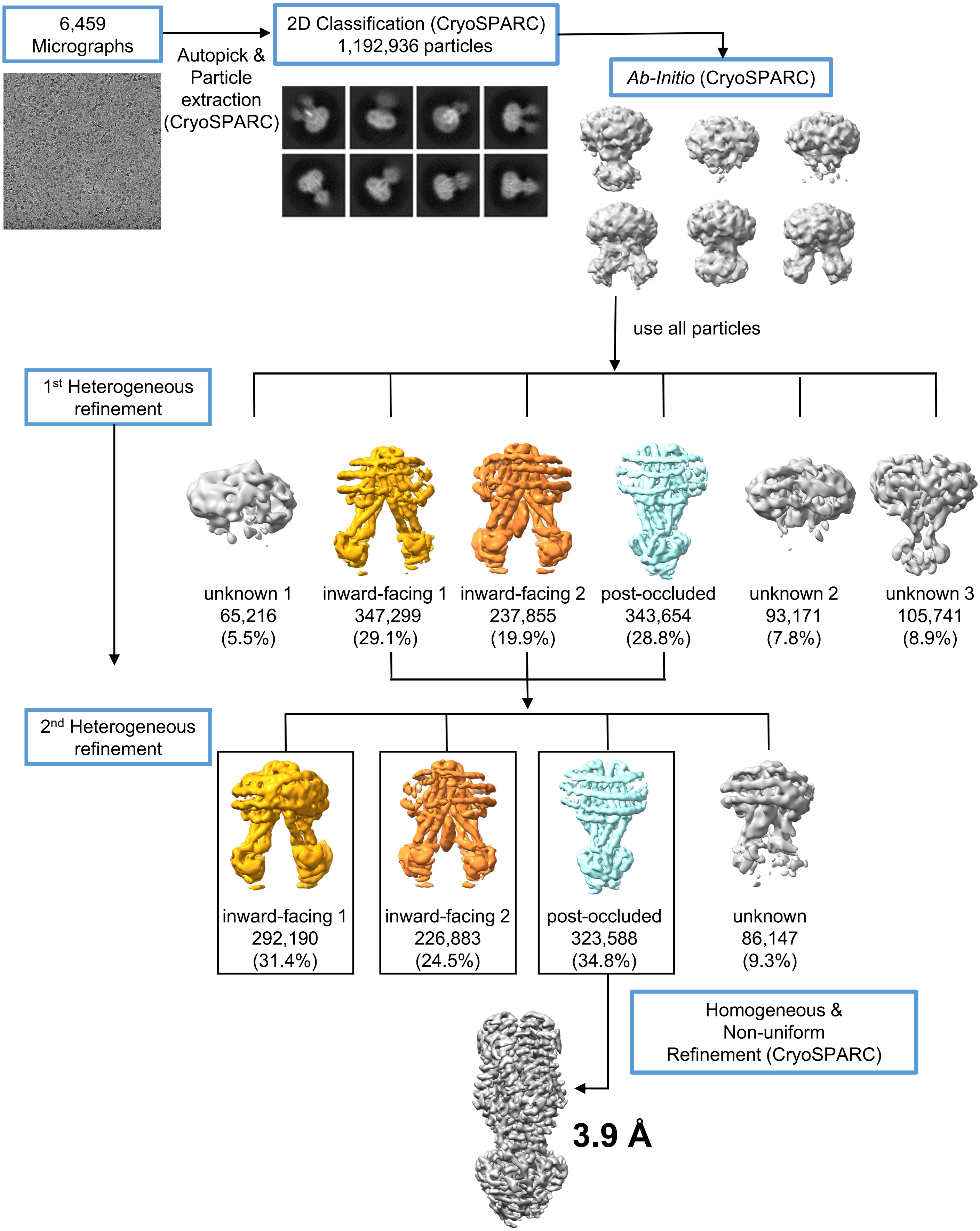

Supplementary Figure 9. Cryo-EM data processing pipeline employed for Mg^{2+}/ADP·VO_4-bound, post-occluded hABCB6^{core}-W546A in nanodiscs. The data processing workflow is presented and major 3D classes shown in Fig. 4d are in black boxes. Please see the Methods section for details.

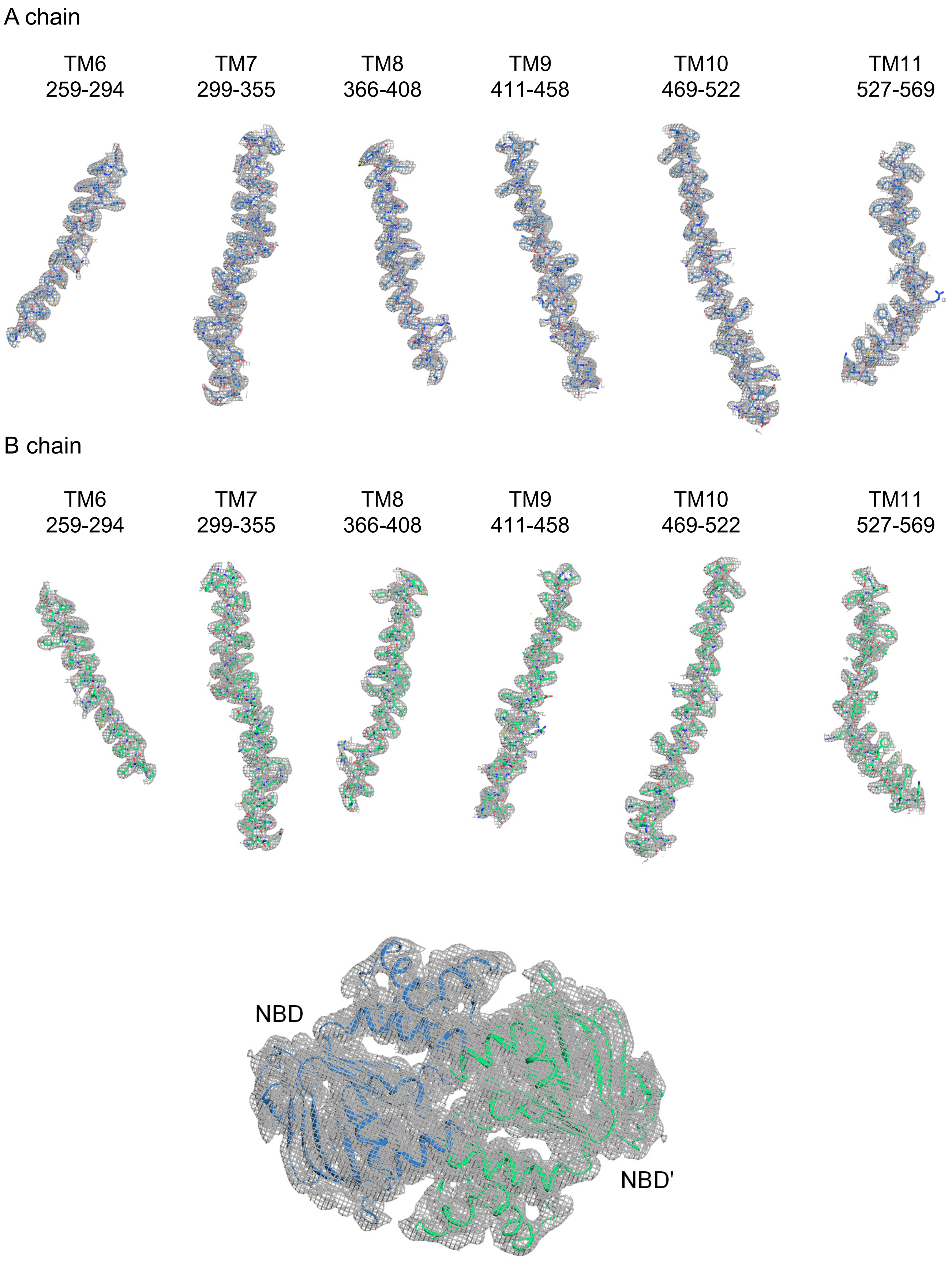

Supplementary Figure 10. Cryo-EM maps of the post-occluded hABCB6^{core}-W546A in complex with Mg^{2+}/ADP·VO_4. Amino acid residues in TMDs are shown, while NBDs are depicted as cartoons. The map is contoured at the 4 σ level.

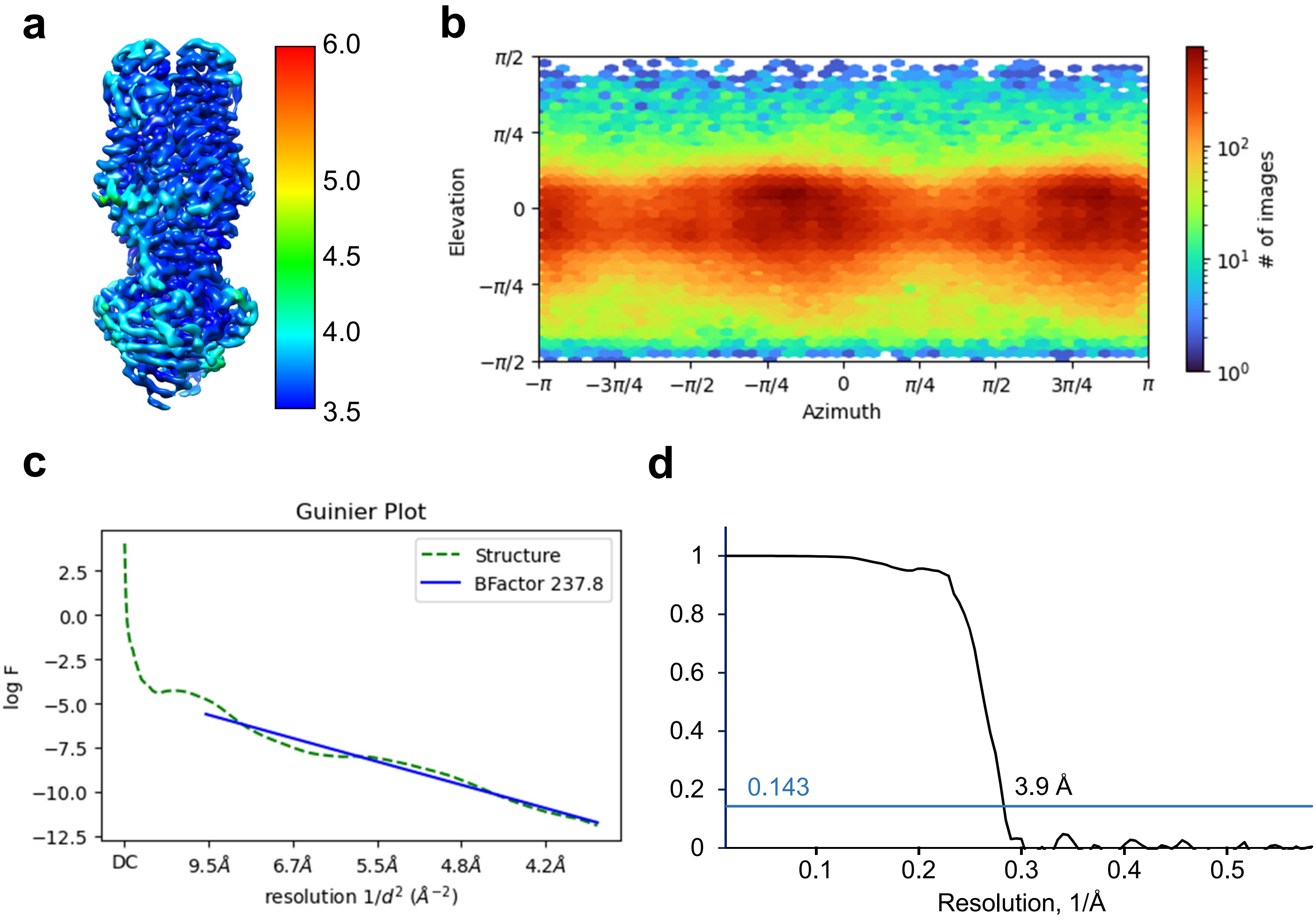

Supplementary Figure 11. Cryo-EM data quality analysis of the post-occluded hABCB6^{core}-W546A in complex with Mg^{2+}/ADP·VO_4. (a) Local resolution of post-occluded hABCB6^{core}-W546A from 3.5 Å (blue) to 6.0 Å (red). (b) Particle orientation distributions in the final 3D reconstruction. (c) Guinier plot for global B-factor estimation. (d) Gold-standard FSC curve. The blue line represents the 0.143 FSC cut-off criterion.

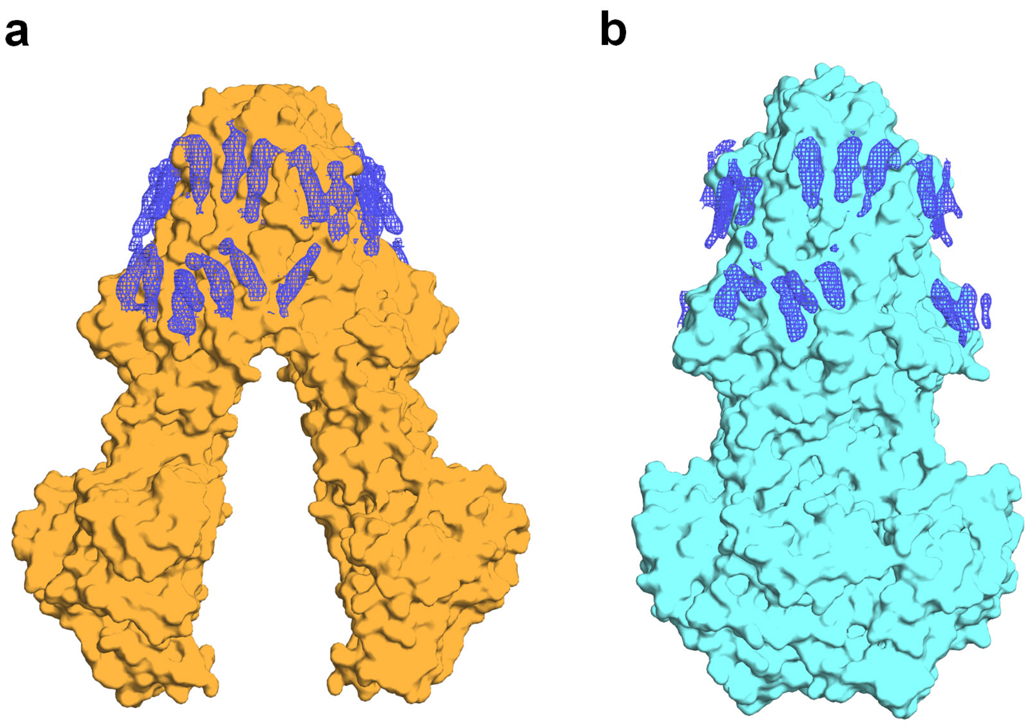

Supplementary Figure 12. Lipid-like densities in the nanodisc-reconstituted hABCB6^{core}-W546A. (a-b) Cryo-EM densities of clearly defined lipid molecules (blue mesh) are displayed at the 5 σ level for both the (a) inward-facing and (b) post-occluded conformations of hABCB6^{core}-W546A.

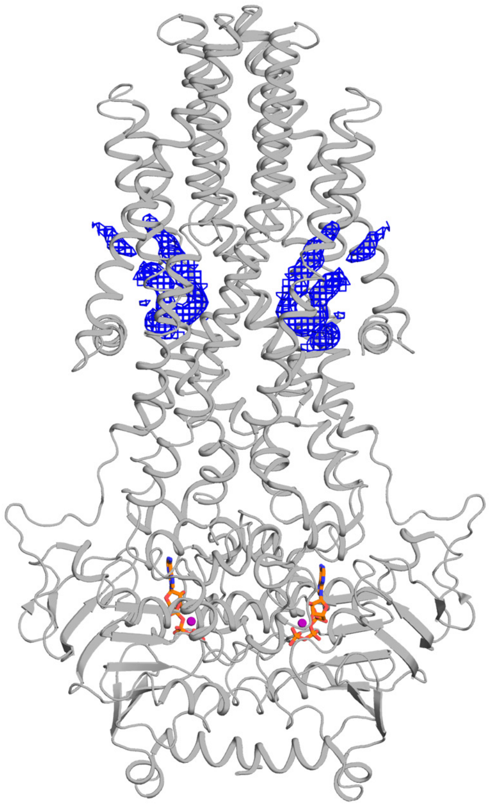

Supplementary Figure 13. Phospholipid density observed in the hydrophobic groove on the TMD surface in the pre-occluded hABCB6^{core} state (PDB ID 7EKL). Cryo-EM maps (blue mesh, EMD-31169) of phospholipids are contoured at the 3 σ level.

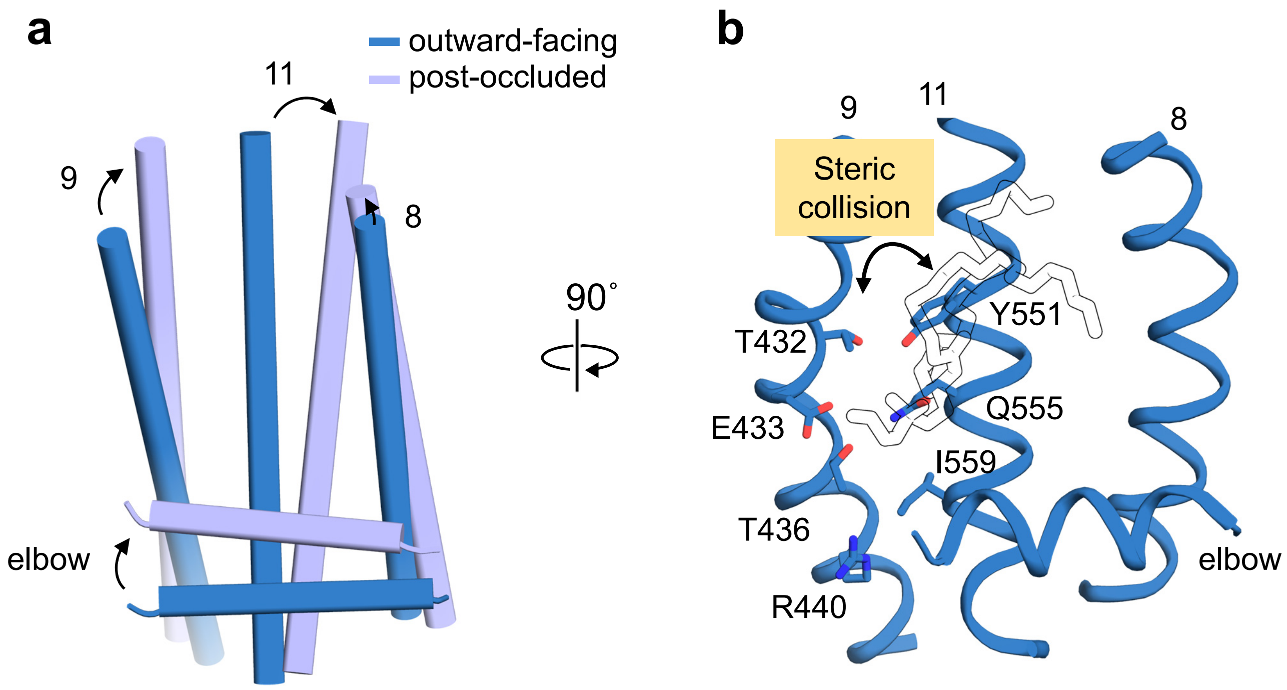

Supplementary Figure 14. Structural comparison of the hydrophobic groove of the TMD surface in outward-facing and post-occluded conformations. (a) Comparison of the conformations of TM helices surrounding the lipid-binding pocket between outward-facing and post-occluded states. Bound POPE in the post-occluded state is omitted for clarity. Black arrows indicate movement of TM helices from the outward-facing to the post-occluded states. (b) No hydrophobic groove is available for lipid binding in the outward-facing conformation. This is likely due to the close proximity of TM 9 and TM 11, which leads to the absence of a lipid-binding pocket in this conformation. The structure of POPE, depicted as empty black sticks, is superimposed on the outward-facing W546A mutant, which likely causes severe steric collisions with neighboring residues.

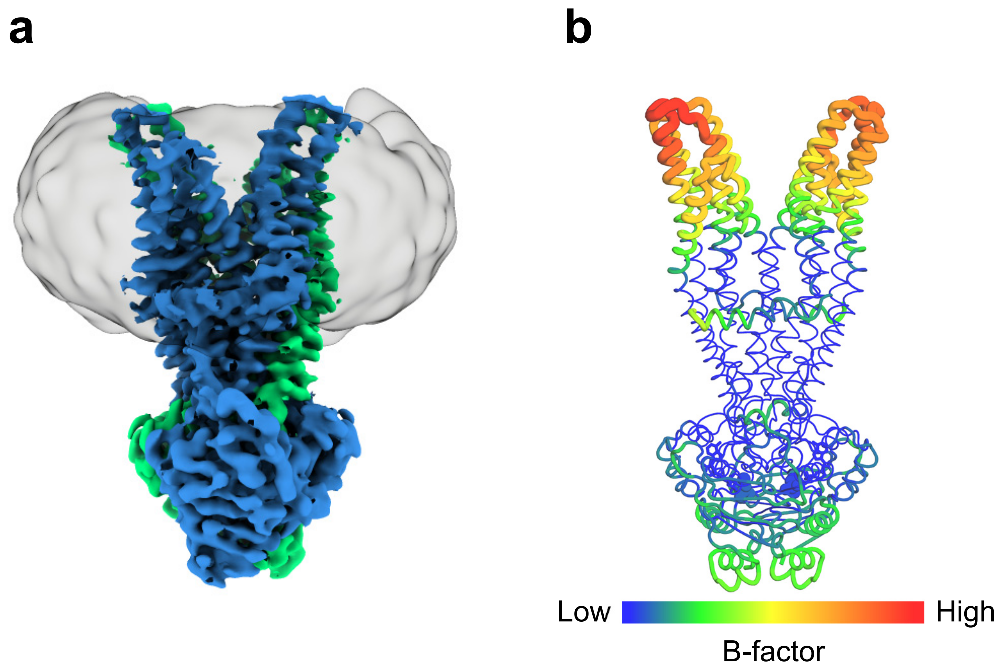

Supplementary Figure 15. Cryo-EM map and B-factor diagram of the hABCB6^{core}-W546A mutant in the outward-facing conformation. (a) Cryo-EM map of outward-facing W546A in complex with Mg^{2+}/ADP·VO_4. Each subunit is colored blue and green, respectively. The detergent micelle is depicted in transparent gray. (b) Front view of the W546A mutant colored according to B-factors.

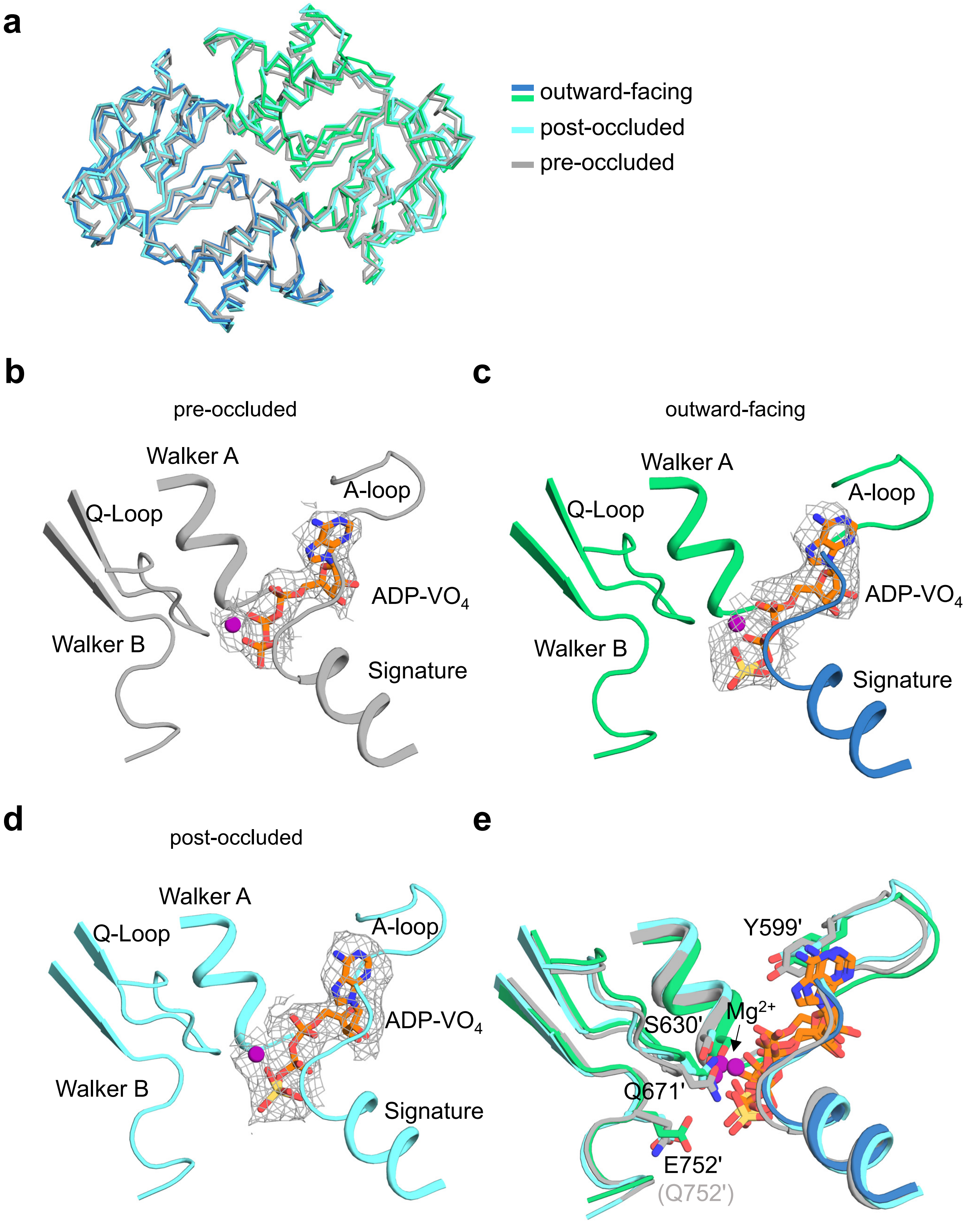

Supplementary Figure 16. Structural comparison of NBDs in outward-facing and occluded conformations. (a) NBD structures of pre-occluded E752Q (PDB ID 7EKL) and outward-facing and post-occluded W546A are superimposed. The Cα r.m.s.d. values between the three structures are all below 1 Å. (b–d) Close-up views of the nucleotide-binding sites in pre-occluded (b, ATP), outward-facing (c, ADP·VO_4), and post-occluded (d, ADP·VO_4) conformations. Mg^{2+} and nucleotides are shown as spheres and sticks, respectively. Cryo-EM maps (gray mesh) of nucleotides are contoured at the 4 σ level. (e) Comparison of nucleotide-binding modes.

Supplementary Table 1. Kinetic values of hABCB6^{core} and W546A proteins determined in detergent micelles and various nanodiscs.

protein	purification	V_{max} (nmol/mg/min)	K_m (μM)
hABCB6^{core}	cymal6/CHS	18.3±0.7	73.2±16.3
	brain lipid nanodisc	96.6±4.0	71.5±10.4
W546A	cymal6/CHS	48.9±1.2	57.6±15.7
	brain lipid nanodisc	39.6±1.5	73.8±16.0
	POPC nanodisc	19.5±0.7	73.8±16.8
	POPS nanodisc	19.0±0.8	66.4±18.0
	POPG nanodisc	21.0±0.7	88.3±15.3
